# The Short-Range, High-Accuracy Compact Pulsed Laser Ranging System

**DOI:** 10.3390/s22062146

**Published:** 2022-03-10

**Authors:** Hongbin Ma, Yuan Luo, Yan He, Shiguang Pan, Lihong Ren, Jianhua Shang

**Affiliations:** 1 School of Information Science and Technology, Donghua University, Shanghai 201620, China; hongbin_ma2021@163.com (H.M.); shiguangpan2021@163.com (S.P.); lhren@dhu.edu.cn (L.R.); 2 Shanghai Institute of Optics and Fine Mechanics, Chinese Academy of Sciences, Shanghai 201800, China; luoyuanhrbeu@163.com (Y.L.); heyan@siom.ac.cn (Y.H.)

**Keywords:** obstacle-avoiding, laser ranging, time-of-flight (ToF), FPGA, temperature correction, gray-value distance correction

## Abstract

A short-range, compact, real-time pulsed laser rangefinder is constructed based on pulsed time-of-flight (ToF) method. In order to reduce timing discrimination error and achieve high ranging accuracy, gray-value distance correction and temperature correction are proposed, and are realized with a field programmable gate array (FPGA) in a real-time application. The ranging performances—such as the maximum ranging distance, the range standard deviation, and the ranging accuracy—are theoretically calculated and experimentally studied. By means of these proposed correction methods, the verification experimental results show that the achievable effective ranging distance can be up to 8.08 m with a ranging accuracy of less than ±11 mm. The improved performance shows that the designed laser rangefinder can satisfy on-line ranging applications with high precision, fast ranging speed, small size, and low implementation cost, and thus has potential in the areas of robotics, manufacturing, and autonomous navigation.

## 1. Introduction

Since the first laser rangefinder with a ruby laser was developed in 1961, laser raging has experienced ongoing development and has since become a mature and well-known active optical distance measuring technology. With the cost reduction of system composition and promising potential technical advances, laser ranging technology is playing a growing role in various applications [1]. Atmospheric studies and surface topography mapping are still mainstream applications of laser ranging [2,3]. In view of the ability to sense surrounding objects in a real-time and high-performance way, laser ranging is bringing about significant changes in modern industry.

Nowadays, the rapid developments in autonomous driving, unmanned aerial vehicles, robotics, and automotive industries have put forward higher requirements for 3-D imaging obstacle avoidance systems [4]. For this purpose, using laser ranging techniques to obtain target distances in different azimuths by controlling the scanning angle of the spatial beam is a tried-and-true solution [5]. For example, the Defense Advanced Research Projects Agency (DARPA) of the U.S. launched ‘the Sweeper’, a small laser radar specially designed for autonomous driving. VLS-128 developed by the Velodyne Company is one of the latest products used for obstacle avoidance in driverless automation with a detection range of 300 m and a minimum angular resolution of 0.11° [6]. With regard to the obstacle avoidance system, there are mainly three parts—those are the beam scanning control, ranging, and imaging. Among them, laser ranging performances—including ranging accuracy, ranging efficiency, and ranging data upload rate—are particularly important [7,8].

The working principles of the laser rangefinder can be divided into time measuring and phase measuring. The type of existing laser rangefinders can be sorted into triangulation mode, pulsed laser mode, frequency-modulated continuous-wave (FMCW) mode, correlation mode, time-correlated single-photon counting (TCSPC) mode, dual-comb mode, and frequency comb interferometry, among others [5]. Each laser ranging method has its merits and shortcomings. The laser rangefinder based on FMCW is realized by a linear modulation of the frequency of a single mode laser and is able to provide the simultaneous measurement of distance and velocity according to the round trip time and the Doppler shift respectively [9,10,11]. By employing the coherent detection, the FMCW laser rangefinder has better ranging precision. However, there is a trade-off between the ranging precision and the cost and complexity of a system (the modulation, optical layout, signal processing, etc.). Related to the TCSPC laser rangefinder, it operates at the ultimate limit of detection sensitivity and can offer a large measurement range at the expense of greater time requirements for statistical analysis [12,13,14]. Meanwhile, the expensive single-photon detector used in the TCSPC laser rangefinder increases the system cost.

Generally speaking, the most common implementation of laser ranging is based on the time-of-flight (ToF). The extremely narrow laser pulse width, excellent laser pulse repetition rate, and high collimation accuracy mean it can measure distance at a higher speed even with a single measurement [15,16]. Most recently, the expanding applications of robotics, manufacturing, and other autonomous navigation technologies also promote higher requirements for short-range rangefinder with high ranging accuracy, high ranging efficiency, high ranging data upload rate, lower cost, and less power consumption [17,18]. The short-range pulsed ToF ranging method is one preferred solution. It presents the advantages of fast response time, high repetition frequency, no continuous modulation, simple system structure, and low power consumption [19]. Furthermore, with the help of a field-programmable gate array (FPGA), the system design will become more simple and cheaper. However, for the time discrimination process of the short-range pulsed ToF rangefinder, the optical detectors with gain, different reflectivity of different targets, and different distances for back reflection, and so on will give rise to timing discrimination errors of the echo laser pulse. As a result, the distance measurement will be inaccurate [20].

In recent years, the system configuration, photodetectors, signal processing method, etc. have been investigated aiming to improve the performance of short-range pulsed ToF laser range finder, and there are a great number of related research reports [21,22,23,24]. The interference from the ambient light is an important factor limiting the actual ranging performance. To reduce the interference from sky background noise and increase the SNR, Ahmed Hassebo et al. used simulations and experiments to optimize the receiver aperture shapes, locations, and sizes in the short range below 2 km [25]. In order to measure the same distance with a lower laser power, the light trapping structures of photodetectors have been studied and the photodetectors with a diameter of 30 μm and a quantum efficiency of more than 55% are developed for short ranges measurement [26]. The lateral propagation of input light into thin layers of silicon and germanium on silicon is improved; correspondingly, the interaction between the detected laser signal and the photodiode are enhanced. Nonetheless, the design of system hardware circuit and the processing of echo signal acquisition data are optimized [27,28]. The signal processing is a necessary and effective academic and technical method to explore better ranging performance.

As previously mentioned, measuring the time interval between transmitted laser pulse and the received laser pulse accurately is a significant guarantee to achieve accurate distance detection for the pulsed ToF laser rangefinder. There is no doubt that the timing discrimination error limits the ranging accuracy. For this reason, a differential hysteresis timing discrimination method is reported in detail [29]. Compared with the traditional leading-edge timing discrimination, this timing discriminator circuit that uses fewer components has better single-shot ranging accuracy. In addition, with the most frequently used detectors in the pulsed ToF ranging system, the avalanche photodiode detector (APD) working in the linear mode and the single-photon detector triggering on its first received photon, Silvano Donati and his co-authors analyze the influence of the timing error as well as the time-walk systematic error, and set up the evaluation method of ranging precision [30]. Their corresponding theory is meaningful for calculating the theoretical ranging precision of the distance measuring instruments, in particular the pulsed ToF laser rangefinder. For nonlinear time walk error, the related compensation method on the basis of double threshold correction is studied. Through setting two threshold points of the rising edge of echo signal pulse and establishing the relationship between these two points and the error, the walk error can be greatly reduced to 0.337 ns [31]. With respect to the improvement of dynamic ranging accuracy, dual-channel timing discrimination by combining constant-threshold timing discrimination method with peak timing discrimination method is proposed [32]. Consequently, the accurate echo timing discrimination is realized, and the improved ranging accuracy of short-range laser rangefinder is within ±30 mm.

In this paper, which aims to improve the ranging accuracy in short-range laser ranging, a compact real-time pulsed ToF laser rangefinder combined with the gray-value distance correction and temperature correction is developed. First, considering the ranging application requirements—such as ranging performances, size, weight, and cost—the laser ranging system design principle and implementation are introduced in detail. Second, in view of the variations of detected target reflectance, photodetector responsibility, and optical propagation properties, the intensity and saturation of echo laser pulse changes, and the rising and falling edges of the echo laser pulse drifts. This broadening of the echo laser pulse seriously restricts the ranging accuracy. Therefore, gray-value distance correction for the timing discrimination is proposed, and the experiments were carried out to verify this method. At a distance of 2355 mm, the results show that the improved distance difference is 16 mm, the standard deviation is up to 4.60 mm, and the ranging accuracy is within ±9 mm. Furthermore, the temperature correction is also adopted to optimize the ranging performances. The specific implementation process and the related experimental results are included. Third, taking a black foam plate with a reflectance of about 0.1 as the detected target, the real-time ranging verification experiments are conducted with the help of gray-value distance correction and temperature correction realized with FPGA. The maximum achievable distance reaches 8.08 m and the corresponding ranging accuracy can be done within ±11 mm. Finally, the conclusion is presented.

## 2. Measurement Principle

**Figure 1:**
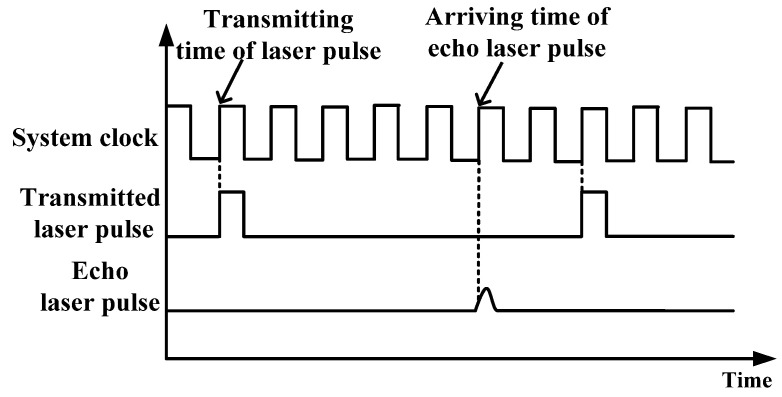
Schematic diagram of pulsed ToF laser ranging.

Pulsed ToF method calculates the distance by measuring the time interval between the transmitted laser pulse and the received echo pulse [33]. The calculation formula of detected target distance is

(1) $$S=\frac{1}{2}ct$$

where *S* is the target distance, *c* is the speed of light in the air, and *t* is the round-trip flight time of laser pulse. For the actual pulsed laser rangefinder, *t* is calculated by recording the number of internal clock counters during the period from the transmission of optical pulse to the receiving of echo signal. As shown in Figure 1, if the internal clock frequency is *f*, the period is *τ*, and the count data from the time of laser emission to the echo signal entering the detector counter is *n*, then equation 1 can be modified as follows,

(2) $$S=\frac{1}{2}cn\tau$$

To sum up, the accuracy of time *t* is related to the internal clock frequency *f* (1/*τ*). The accuracy of *t* then affects the ranging accuracy (Δ*S*). The ranging accuracy (Δ*S*) can be calculated by,

(3) S=nΔS

$$\Delta S=\frac{1}{2}c\tau =\frac{c}{2f}$$

## 3. System Design

Different ranging methods have their own advantages and scopes of application. The laser ranging system of this project is mainly used for short ranges (<8 m), such as mobile robot obstacle avoidance and intelligent storage, which requires high precision, fast ranging speed, good real-time performance, and low implementation cost. Considering the application requirements, performance, and cost of the system, the pulsed ToF distance measuring method is adopted for our ranging system. In order to improve the ranging accuracy, the temperature correction and gray-value distance correction are carried out by means of FPGA programming.

**Figure 2:**
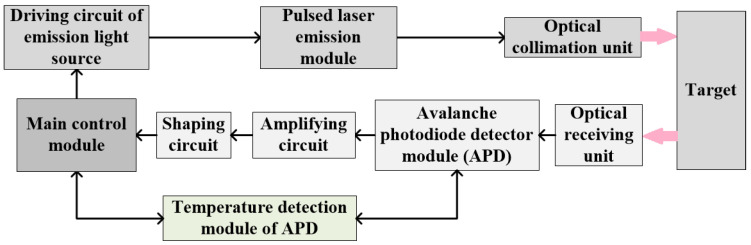
Structure diagram of a short-range pulsed laser ranging system.

Figure 2 shows the structure of the short-range pulsed laser ranging system based on the pulsed ToF ranging method. The laser ranging system is mainly divided into three parts. One is the main control module implemented on FPGA chip, which is responsible for the control, data processing, and distance calculation of the whole ranging system. Considering the demand of high-speed signal acquisition and high operation speed of the designed system, the Cyclone IV series FPGA chip of Altera is used as the main control chip. The supporting circuit and program are designed on the chip to realize the software and hardware design of a pulsed laser ranging system. The second is the pulsed laser emission module. The laser is the key part which affects the performance of the ranging system. The driving circuit is designed according to the characteristics of the laser. The repetition frequency and pulse width are set in the FPGA program. The optical collimation unit is used to reduce the energy divergence. The third is the echo signal receiving module composed of an avalanche photodiode detector (APD). The external optical signals are converged by APD through an optical receiving system. The APD generates the response current in response to the received optical signal, and after the amplification and shaping circuit, the signal is collected by the high-speed acquisition module of FPGA. The distance information carried by the signal is processed and calculated by the internal program. In addition, due to the temperature characteristics of APD and the influence of temperature on electronic devices, the temperature detection module should be set separately, and the APD reverse bias voltage should be set according to the temperature feedback. The actual ranging results must also be corrected by temperature correction.

## 4. Numerical Calculation

Owing to the transmitted laser pulse divergence of the laser rangefinder, there is a laser facula on the detected target surface. Depending on the difference between the area of the laser spot and that of the target, the laser ranging equation can be sorted as the range equation for the small diffuse reflecting target and the range equation for the large diffuse reflecting target. In terms of the long-range distance measurement, where the area of the laser spot is larger than that of the detected target, the detected target is treated as the small diffuse reflecting target and the photodetector detectable power *P_r_* is in accordance with Equation (4) [34,35].

(4) $$P_{r}=P_{t}\frac{A_{r}\sigma }{4\pi \theta_{t}^{2}R^{4}}T_{t}T_{r}T_{a}^{2}$$

where *P_t_* is the transmitted laser power, *A_r_* is the area of receiver, *σ* is the radar cross section of target, *θ_t_* is the probing beam divergence, *R* is the detected distance, *T_t_* is the optical efficiency of the transmitting system, *T_r_* is the optical efficiency of the receiving system, and *T_a_* is the one-way transmission efficiency through the atmosphere.

Otherwise, with regard to short-range distance detection, the laser spot is smaller compared with the efficient reflecting surface of the detected target, and the detected target is typically noncooperative and can be assumed as a Lambertian target. In this case, the radar cross-section of detected target *σ* can be described as Equation (5). Accordingly, the photodetector detectable power *P_r_* is expressed by Equation (6) [36,37].

(5) $$\sigma =4\rho R^{2}\theta_{t}^{2}cos\varphi$$

(6) $$P_{r}=P_{t}\frac{A_{r}\rho cos\varphi }{\pi R^{2}}T_{t}T_{r}T_{a}^{2}$$

where *ρ* is the reflectance of the detected target, and *φ* is the included angle between probing beam optical axis and target normal direction.

For the designed short-range laser rangefinder, the detected target belongs to the large diffuse reflecting target, and the maximum detectable distance *R_max_* is achieved when the photodetector detectable power *P_r_* reaches its minimum value *P_min_*. The *R_max_* one critical range index of a laser rangefinder can be calculated using Equation (7). Obviously, the lower the minimum detectable power of the photodetector (*P_min_*), the farther the maximum detectable ranging distance *R_max_* of the designed laser rangefinder.

(7) $$R_{max}=\sqrt{P_{t}\frac{A_{r}\rho cos\varphi }{\pi P_{min}}T_{t}T_{r}T_{a}^{2}}$$

The optical receiving module receives all optical signals without difference during the practical distance measurement. Therefore, the *P_min_* heavily depends on the signal-to-noise ratio (*SNR*) which can be calculated by [38],

(8) $$SNR=\frac{I_{s}}{I_{n}}$$

$$I_{n}^{2}=I_{ns}^{2}+I_{nb}^{2}+I_{nd}^{2}+I_{l}^{2}$$

where *I_s_* is the signal photocurrent of the APD, *I_n_* is the noise current which is mainly composed of signal light shot noise current (*I_ns_*), background light shot noise current (*I_nb_*), dark current shot noise current (*I_nd_*), and thermal noise current (*I_l_*). The background shot noise current is directly related to the background light power (*P_b_*) which can be obtained by [35],

(9) $$P_{b}=\frac{\pi }{16}T_{r}\rho \theta_{r}^{2}d_{r}^{2}\Delta \lambda \left[T_{a}\rho H_{\lambda }cos\theta cos\varphi +\pi N_{\lambda }\left(1-T_{a}\right)\right]$$

where *θ_r_* is the receiving field angle, *d_r_* is the receiving lens diameter, Δ*λ* is the spectral bandwidth of the narrow-band filter, *H_λ_* is the spectral irradiance of the sun light to the ground, *θ* is the angle between the target surface normal and the sun light, and *N_λ_* is the radiance of the solar spectrum scattered by the atmosphere.

**Figure 3:**
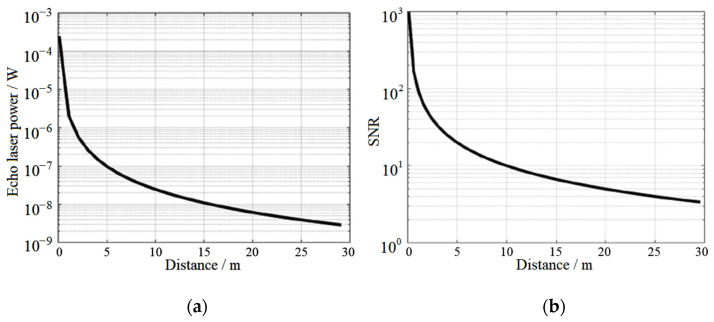
Echo laser power (**a**) and SNR (**b**) with the detection distance.

The related design parameters of the system are shown in Table 1. The one-way atmospheric transmittance is approximately 0.99, and the corresponding value of solar irradiance is 748.5 W/m^2^ (@ 905 nm). 𝜃 is the angle between the target surface normal and the sun light, and the estimated value is 44° for the simulation calculation. 𝜑 is the angle between the receiving optical axis and the target surface normal and the cos𝜑 is the approximate value of 1. Generally, the reflectivity of ground objects is from 0.1 to 0.7. The smaller the reflectivity of the target is, the closer the ranging distance of the system is. In our calculation, the reflectivity of the target is taken as 0.1 which means the calculation result is the conservative longest ranging distance. Combining Equations (7)–(9) and Table 1, the relationship between the received echo optical power, *SNR*, and detection distance can be obtained. As shown in Figure 3, for the designed laser ranging with system parameters shown in Table 1, the *SNR* equals to 10 is considered as a critical point to distinguish the signal from the noise. In this way, the detection distance about 10 m at this point shows the maximum detectable distance of the designed system with the target reflectivity of 0.1.

## 5. Experiment Results and Analysis

The system error will affect the ranging performance seriously. Based on the principle of ToF, the system error mainly comes from the measurement accuracy of flight time when the influence of atmospheric environment on the speed of light is ignored. The system error can be divided into the random error and the system static error.

The random error mainly includes output jitter of threshold comparison, timing error, circuit noise, and so on, and can be reduced by selecting high-precision devices. For example, in the shaping conversion circuit, the output jitter can be reduced by converting the output voltage into low voltage differential signal (LVDS). For the random error caused by timing, the FPGA with a high-precision timing module is an attractive option. In addition, improving the noise suppression ability of circuits is an effective way to reduce the random error.

Concerning the static error, it mainly includes the time-delay error, temperature drift error, and echo pulse width drift error. Specifically, the invariable time-delay error during ranging results from the designed laser ranging system itself. One is the delay between the theoretical laser transmitted time controlled by FPGA and the actual laser transmitted time, the other time delay comes from the target’s range information extraction hardware circuitry. Since the time delay mentioned above is fixed, it can be corrected. The temperature drift error is not only from the APD temperature drift, but also from the semiconductor laser source and other electronic devices. The echo pulse width drift error is caused by the target reflectivity difference. The detected target with different reflectivity leads to the change of reflected laser pulse intensity and saturation. Correspondingly, the rising edge, descending edge, and reflected laser pulse width will drift at the same time and the ranging performances degrade definitely. Therefore, the gray-value distance correction and temperature correction described as follows are proposed so as to effectively eliminate the static error in the designed laser ranging.

### 5.1. Gray-Value Distance Correction

The pulsed ToF laser rangefinder obtains the detected distance by measuring the time interval between the transmitted laser pulse and the received laser pulse. The transmitting time of the laser pulse is same. For the same detected distance, the measured time interval should also be same. However, resulting from the variations of detected target reflectance, photodetector responsibility, and optical propagation properties and so on, even with the same detected distance the intensity and saturation degree of echo laser pulse changes which leads to the drift of rising and falling edge and the widening of echo laser pulse width in time domain [39,40]. Consequently, the timing discrimination is inaccurate and the corresponding ranging accuracy cannot be guaranteed. Regarding future short-range dynamic ranging, with both the laser rangefinder and the detected target moving relatively, this issue will become more serious.

**Figure 4:**
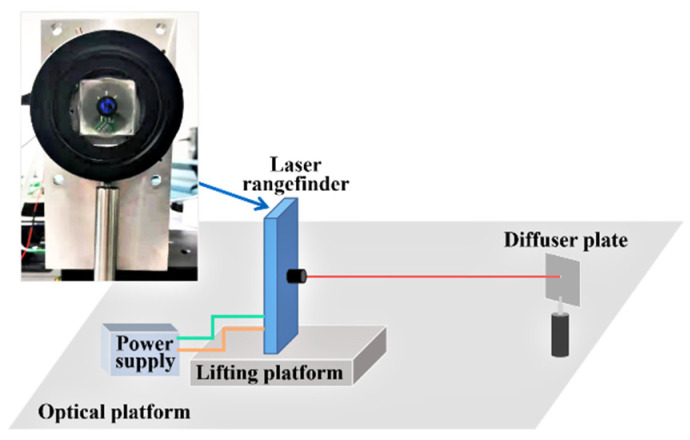
The gray-value distance correction experimental platform.

With the purpose of establishing the influence relationship between the echo laser pulse width and its corresponding distance offset, the gray-value distance correction experimental platform shown in Figure 4 is built. In the gray-value distance correction process, a series of diffuser plates with different reflectance are selected as the detected targets and are put on a fixed position one by one. The diffuser plate is a kind of typical Lambertian reflector with a reflectance from 2% to 99% in the wavelength range between 250 nm and 2500 nm and it is the common and standard laser ranging target used in the laser ranging experiments. They can represent the detected targets with different reflectances and are useful in verifying the experimental results.

**Figure 5:**
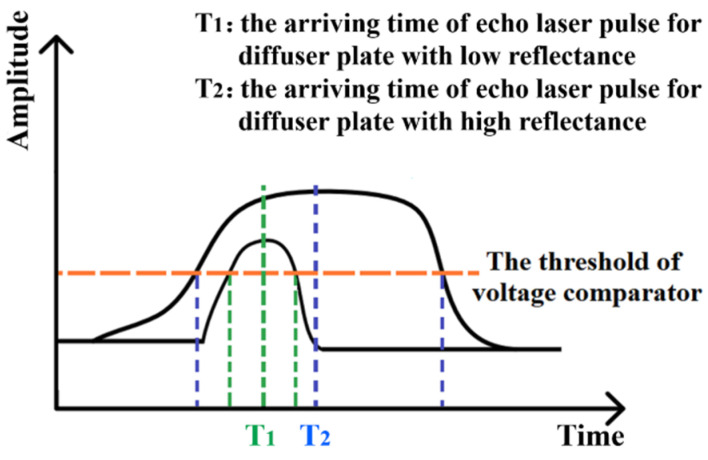
Arriving time of the echo laser pulse.

In the signal processing part of the designed system, using the voltage comparator with a fixed threshold, the echo laser pulse is converted into the echo pulse signal and its arriving time is determined. Just like what shown in Figure 5, even for the same fixed-point laser ranging, the echo laser pulse arriving times T_1_ and T_2_ for diffuser plates with low reflectance and high reflectance are different from each other. Consequently, the timing discrimination and the time interval are not accurate and there will be the distance offset compared with the actual distance. For the same fixed-point ranging tests, with help of the diffuser plate with different reflectance, the gray-value distance correction are conducted and the relationship between the echo laser pulse width and the corresponding distance offset is established. On this basis, the correction parameters and function are obtained and the distance extraction algorithm is corrected. Following, the optimized distance extraction algorithm is programmed through FPGA so as to counteract the effects of the timing discrimination error in real time and improve the ranging accuracy.

**Figure 6:**
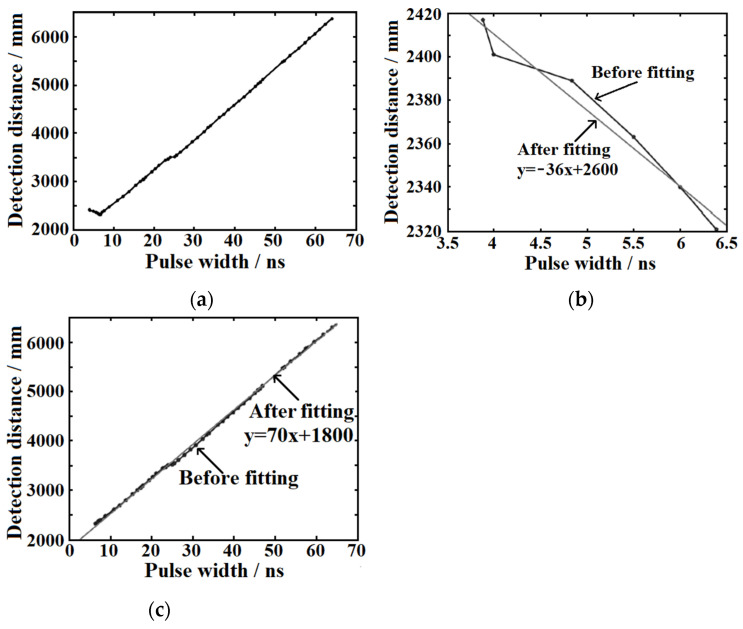
For one fixed-point ranging test, the relationship between detected distance and echo laser pulse width (**a**) and its fitting function (**b**,**c**).

According to 54 groups of test results in the first round, the fitted function curve is shown in Figure 6a. The pulse width represents the actual echo signal pulse width. By analyzing the fitting function curve shown in Figure 6a, it can be seen that there is an obvious turning point in the curve of the echo signal pulse width and ranging distance when the pulse width is equal to 6.5 ns. Taking this point as the inflection point, the fitting curve can be approximately separated into two linear sections (Figure 6b,c). The two fitting function curves are linear functions and the corresponding linear function coefficients of −36 and 70 are selected as the correction coefficients, besides that, the actual echo signal pulse width of 6.5 ns is taken as the correction inflection point.

**Figure 7:**
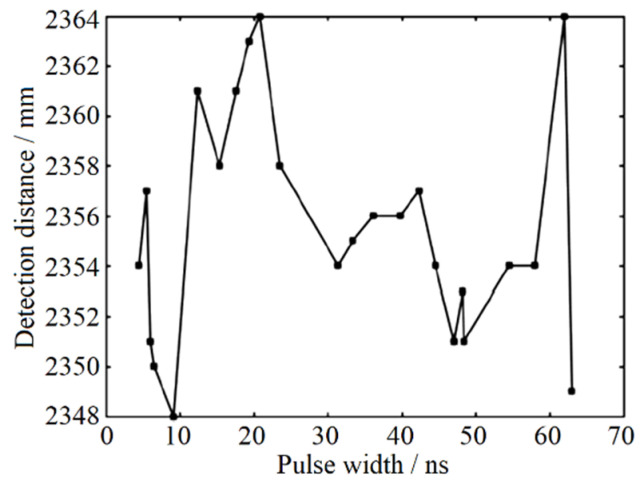
Ranging results after gray-value distance correction.

Seeking to reach the maximum effect of the gray-value distance correction with more accurate correction coefficients, the correction experimental process is carried out repeatedly. Depending on the primary correction coefficient mentioned above, the correction coefficients are further modified and the inflection point distribution is refined again. Through repeated tests and corrections, the final result of fixed-point ranging test after gray-value distance correction is shown in Figure 7. Here, the actual detected distance is 2355 mm. With the help of gray-value distance correction, the distance difference of the designed pulsed laser rangefinder is 16 mm, the range standard deviation is 4.60 mm, and the ranging accuracy is no more than ±9 mm.

It should be noted that whatever the cause (e.g., the real-time gain variation of detector, the optical propagation properties, or others), if the variation causes the widening of echo laser pulse width, this gray-value distance correction method will be an effective means to improve the ranging accuracy.

### 5.2. Temperature Correction

The operating characteristics of semiconductor lasers and other electronic devices in ranging system are easily affected by surroundings. The temperature drift also affects the ranging performances of the system. Therefore, it is necessary to test the relationship between temperature and distance for temperature compensation and correction. Here, the fixed target is selected for testing to obtain the relationship between temperature change and fixed-point distance detection.

**Figure 8:**
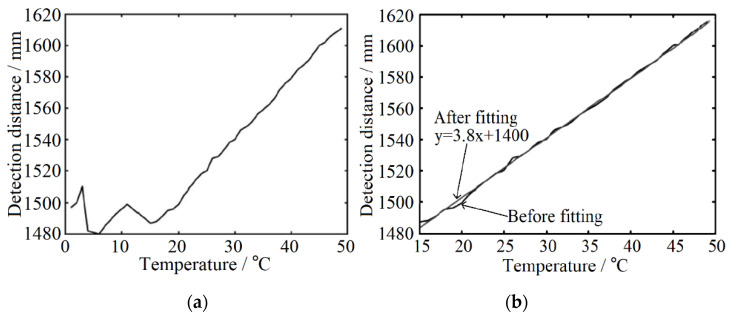
Variation curve of ranging results with temperature (**a**) and its fitting results (**b**).

**Figure 9:**
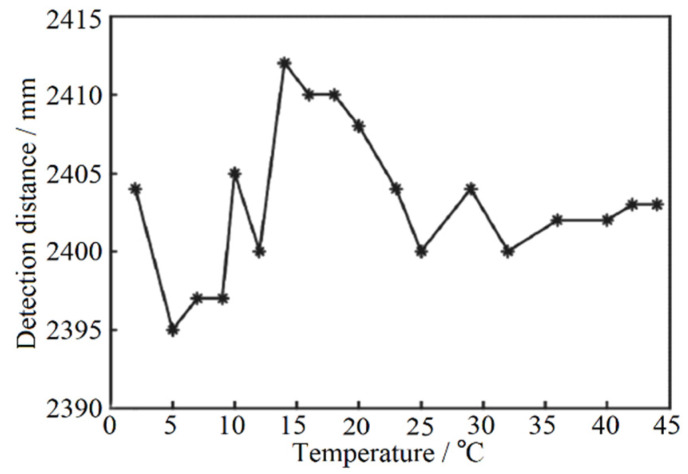
Ranging results after temperature correction. (* is the actual distance measurement result of the designed laser rangefinder).

The function curve fitted according to the statistical test results is shown in Figure 8a. When the temperature is lower than 16 °C, the distance offset is small and non-linear with the change of temperature. However, when the temperature is higher than 16 °C, the distance measurement result has an obvious linear relationship with temperature and the offset is large. Therefore, this experiment focuses on correcting the influence of temperature on ranging above 16 °C. Similar to the gray-value distance correction, the temperature distance curve is fitted and the temperature inflection point is 16 °C. The fitting function is shown in Figure 8b and the preliminary correction coefficient is 3.8. According to the conversion relationship of FPGA program algorithm of temperature correction module, the correction coefficient needs to be magnified by four times, that is, the correction coefficient is set as 15, and the actual distance of the fixed point is taken as the guideline for the initial correction. The correction coefficient can be set to 0 when the temperature is lower than 16 °C. In the process of experiment, the correction coefficient is modified based on the primary correction coefficient to improve the accuracy of ranging continuously. After 20 groups of repeated tests, the final temperature correction test results are shown in Figure 9. The accuracy of the system ranging is ±10 mm and the standard deviation is 4.70 mm at the condition of the fixed-point distance is 2402 mm.

### 5.3. Longest Ranging Distance and Ranging Accuracy Experiment

**Figure 10:**
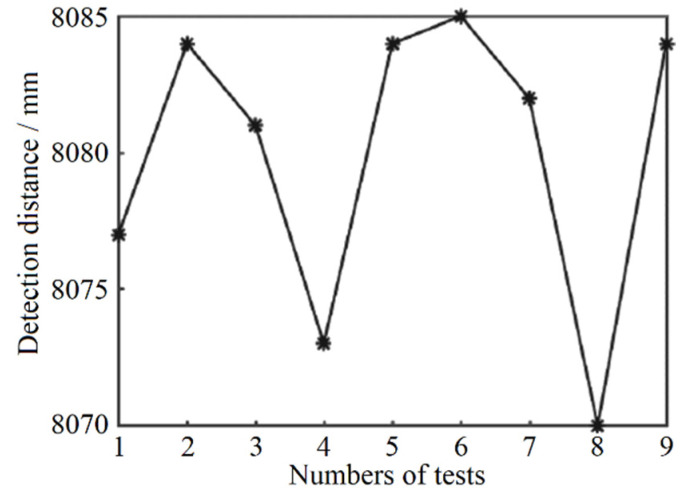
The achievable longest ranging distance. (* is the actual distance measurement result of the designed laser rangefinder.)

The maximum ranging range simulation result is 10 m with the target reflectivity parameter of 0.1. After completing the gray-value distance correction and temperature correction of the pulsed laser ranging system, the verification experiment of the longest detection distance was carried out. In the experiment, a black foam plate with a reflectance of about 0.1 is chosen as the measured target. The longest distance of the signal is obtained by moving the black foam plate position. Finally, nine sets of test data are obtained and shown in Figure 10. The result shows that the maximum distance of the system is 8.08 m.

The final ranging accuracy with different distances of the system was also verified in the experiment. The experiment conditions are similar to the longest distance experiment and the different distances are obtained by moving the black foam plate. The experiment results are shown in Table 2. From 1 m to 8 m, the deviation between the distance measurement results and the actual distance is less than ±11 mm. Combined with the gray and temperature correction, the ranging accuracy of the laser rangefinder designed in this paper is within ±11 mm.

## 6. Conclusions

A short-range, high-accuracy, and compact real-time pulsed ToF laser ranging system combined with the gray-value distance correction and temperature correction is developed. In terms of the timing discrimination error resulting from the variations of detected target reflectance, photodetector responsibility, and optical propagation properties, the proposed methods of gray-value distance correction and temperature correction are capable of improving the ranging accuracy effectively. Through the ranging verification experiments, the results show that—for a target with a reflectivity of 0.1—the maximum achievable ranging distance is up to 8.08 m and the corresponding ranging accuracy can be done within ±11 mm.

## Figures and Tables

**Table 1:** Main parameters of the laser ranging system.

Parameter		Value
Laser	Wavelength	905 nm
	Repetition frequency	1 MHz
	Average transmitted laser power, *P_t_*	1 W
	Beam divergence angle (parallel) θ//	11°
	Beam divergence angle (perpendicular) θ⊥	23°
	Working voltage	11 V
	Duty cycle	0.1%
APD	Responsivity	58 A/W
	Noise equivalent power	2–14 W
	Bandwidth	150 MHz
	Receiving aperture	26 mm
	Noise figure	3
	Filter bandwidth, Δ*λ*	20 nm
Solar irradiance @ 905 nm		748.5 W/m^2^
Optical efficiency of the receiving system, *T_r_*		0.15
Optical efficiency of the transmitting system, *T_t_*		0.975
Receiving field angle, *θ_r_*		0.02
One-way atmospheric transmission efficiency, *T_a_*		0.99
Included angle between target surface normal and the sun light, 𝜃		44°
Included angle between probing beam optical axis and target normal direction, *φ*		cosφ ≈ 1
Reflectance of the detected target, *ρ*		0.1

**Table 2:** Experiment results of the ranging accuracy.

Ranging Value (m)	Actual Value (m)	Deviation (m)
1.012	1.005	−0.007
2.008	2.006	−0.002
3.010	3.006	−0.004
4.004	4.000	−0.004
5.021	5.010	−0.011
6.024	6.019	−0.005
7.000	7.000	0.000
8.000	8.006	0.006

## Data Availability

The data that support the findings of this study are available from the corresponding author upon request.
